# Vasospasm in Cerebral Inflammation

**DOI:** 10.1155/2014/509707

**Published:** 2014-12-29

**Authors:** Michael Eisenhut

**Affiliations:** Luton and Dunstable University Hospital NHS Foundation Trust, Lewsey Road, Luton LU4ODZ, UK

## Abstract

All forms of cerebral inflammation as found in bacterial meningitis, cerebral malaria, brain injury, and subarachnoid haemorrhage have been associated with vasospasm of cerebral arteries and arterioles. Vasospasm has been associated with permanent neurological deficits and death in subarachnoid haemorrhage and bacterial meningitis. Increased levels of interleukin-1 may be involved in vasospasm through calcium dependent and independent activation of the myosin light chain kinase and release of the vasoconstrictor endothelin-1. Another key factor in the pathogenesis of cerebral arterial vasospasm may be the reduced bioavailability of the vasodilator nitric oxide. Therapeutic trials in vasospasm related to inflammation in subarachnoid haemorrhage in humans showed a reduction of vasospasm through calcium antagonists, endothelin receptor antagonists, statins, and plasminogen activators. Combination of therapeutic modalities addressing calcium dependent and independent vasospasm, the underlying inflammation, and depletion of nitric oxide simultaneously merit further study in all conditions with cerebral inflammation in double blind randomised placebo controlled trials. Auxiliary treatment with these agents may be able to reduce ischemic brain injury associated with neurological deficits and increased mortality.

## 1. Introduction

Cerebral vasospasm has been defined as “the reversible reduction in calibre of the lumen of a conducting artery in the subarachnoid space” [1]. The reduction in calibre refers to the appearance of cerebral arteries on an angiograph. Small diameter cerebral arteries play important roles in the autoregulation of cerebral blood flow, matching local blood supply in the brain to neuronal activity. Although angiography, which can assess arteries >1 mm in diameter, has long been the standard to diagnose vasospasm, constriction of smaller cerebral arteries may also contribute to ischaemia and remain undetectable by angiography. Lindegaard developed blood velocity measurements using the noninvasive method of transcranial Doppler ultrasound for definition of cerebral vasospasm [2]. An inverse relation between vessel diameter on angiography and cerebral blood flow velocity (CBFV) on transcranial Doppler sonography has been found and there is considerable evidence that these alterations reflect changes in calibre of the insonated vessels as a result of transient or persistent narrowing. A ratio of >3 in middle cerebral artery flow to extracranial internal carotid artery flow was found to be diagnostic of vasospasm [3]. Transcranial Doppler ultrasound was determined in a meta-analysis as being approximately 67% sensitive for middle cerebral artery spasm and 42% sensitive for anterior cerebral artery spasm [4]. If severe enough vasospasm can lead to cessation of distal blood flow and if present for a sufficient duration and extent it can cause cerebral infarction. Positron emission tomographic studies showed that ischemic deficits from vasospasm were associated with regions of reduced blood flow [5]. None of the methods mentioned may however yield features of vasospasm if this affects transiently precapillary sphincters only.

The risk of infarction depends on adequacy of collateral blood supply, cardiac output, blood pressure, and intracranial pressure. In the context of cerebral inflammation many different factors influence cerebral blood flow. They include inflammatory hyperaemia, increased intracranial pressure, arterial CO_2_, body temperature, mean arterial pressure, the use of mechanical ventilation, and whether patients are sedated during procedures [6]. Physiological regulation of cerebral perfusion is dominated by pressures of CO_2_ and O_2_ in the cerebral circulation. Cerebral vasodilatation in response to hypercapnia is dependent on formation of nitric oxide, a mediator released in inflammation [7]. After release by endothelium NO stimulates soluble guanylate cyclase in vascular muscle resulting in an increase in the intracellular concentration of guanosine 3′,5′-cyclic monophosphate (cGMP) resulting in relaxation. NO is generated from L-arginine by NO synthase. It is the endothelial NO synthase which regulates cerebral blood vessel tone under basal conditions [7].

This review includes studies investigating outcomes like radiological or clinical evidence for focal cerebral ischaemia and infarction. Cerebral vasospasm is a potentially preventable and treatable cause of ischemic cerebral damage. A current lack of established treatment options was the motivation for this review of cerebral vasospasm in conditions with inflammation of the brain. The objective was to investigate whether there is evidence of cerebral vasospasm in all conditions associated with cerebral inflammation and whether there are common pathways to vasospasm in all conditions with cerebral inflammation pointing to common therapeutic options, which may have been explored in one type of cerebral inflammation but should be evaluated in all others with the aim of preventing irreversible cerebral damage, thus reducing morbidity and mortality.

## 2. Methods

The literature search for this review used the database PubMed (search from beginning of database until 31 July, 2014) entering search terms “cerebral vasospasm,” “inflammation,” “meningitis,” “brain injury,” “cerebral malaria,” “subarachnoid haemorrhage,” “ tumor necrosis factor,” “endothelin,” “nitric oxide,” “arachidonic acid,” “rho-kinase,” “fasudil,” “erythropoietin,” “calcium channel blockers,” “statins,” “tirilazad,” and “magnesium.” References of articles were screened for relevance and included where appropriate.

## 3. Vasospasm in Cerebral Infection

### 3.1. Bacterial Meningitis

Tuberculous meningitis is the form of cerebral inflammation in which vasculopathy has been the subject of most detailed studies starting in the 19th century [8]. Radiologically identifiable cerebral infarctions are common in tuberculous meningitis and are present in 20% at initial assessment and in a further 10% they occur after start of treatment [9]. Three main pathologies have been proposed: vasculitis most prominent in vessels passing through the basilar exudate, proliferative changes with intimal thickening with resultant stenosis or occlusion, and necrotizing vascular lesions. The fibrinoid necrosis in supplied tissue found in the absence of blood vessel wall infiltration raised the possibility of vasospasm as an additional feature of the vasculopathy. In some instances infarction was found to have occurred without vasculitic changes or thrombosis compatible with vasospasm.

Supportive of an important role for vasospasm in the pathology of tuberculous meningitis was also the poor correlation between angiographic findings and infarction [10, 11]. The first direct evidence for vasospasm in bacterial meningitis was established in a case of* Haemophilus influenzae* meningitis [12]. A prospective investigation of 22 adults with bacterial meningitis by transcranial Doppler sonography related the degree of arterial narrowing to outcome: Low Glasgow coma scales (<7) on admission, focal cerebral ischaemic deficits, and seizures were more frequent in patients with cerebral blood flow velocity >210 cm/s compatible with vasospasm. Fatal outcome was only found with this high velocity. Patients with minor or no vascular involvement had less prominent initial impairment of consciousness [13]. Other investigations confirmed features of cerebral vasospasm in bacterial meningitis [14]. Patients with features of vasospasm had significantly increased concentrations of IL-1 and IL-6 but not endothelin-1 (ET-1) or thromboxane levels in the CSF. Underlying mechanisms have been explored in a rat model, where cerebral blood flow as measured by injection and recovery of microspheres from the brain tissue was found to be reduced in pneumococcal meningitis. The cerebral blood flow could be restored by the endothelin-1 antagonist bosentan with a reduction in cortical cerebral injury [15]. Strong evidence of a protective role of nitric oxide against vasospasm in bacterial meningitis was derived from an animal model of neonatal meningitis where inhibition of NO synthase worsened outcome in some models of bacterial meningitis, leading to local cerebral ischaemia [16].

### 3.2. Cerebral Malaria

There is accumulating evidence for disseminated vasospasm triggered by inflammatory mediators produced locally in the vascular bed in response to the sequestered infected red blood cells in cerebral malaria (CM). Reversible vasospasm could explain the rapid onset but also often rapid and complete resolution of symptoms of CM. Vascular obstruction by clumps of infected blood cells, platelets, and uninfected red blood cells tightly adhering to endothelium is less likely to be rapidly reversible without leaving irreversible neurological damage, which occurs in a minority of patients with CM.

In a seminal study of the morphology of arterioles in the brain of patients who died of cerebral malaria Polder et al. demonstrated as early as 1990 that there is evidence of spastic contraction of arterioles with folding of the whole vessel wall and of the astrocytic plasma membrane and glial basement membrane with total occlusion of the lumen [17]. There was light microscopic interruption of the flow at the arteriolar level resulting in irregular perfusion of the capillaries. Numerous cerebral capillaries were entirely empty. Recent in vivo studies on retinas in 34 patients with CM by fluorescein angiography showed the abrupt pruning of larger arterioles with normal perfusion right up to the point of nonperfusion suggesting an arterial vasospasm. Normal angiograms in six patients including two with neurological sequelae indicated a lack of longstanding vasoocclusion. This observation was compatible with a transient vasospasm [18]. Evidence for a temporary vasoocclusion as seen in a vasospasm was also the macular retinal whitening, which occurred without matching zones of capillary nonperfusion at the time of the angiogram in 7/34 patients including four for whom the angiogram was delayed >24 hours after admission.

There was no evidence for a reduced global cerebral blood flow on extracranial basal artery Doppler measurements. High CBFV was recorded in 30% of children with CM during admission [19]. This suggested that ischemic events had passed by the time the angiography was performed as would be seen in a transient vasospasm. In CM cerebral arteriolar vasospasm may be mediated by tumor necrosis factor (TNF). The degree of TNF elevation in the cerebrospinal fluid of children with CM has been associated with risk of neurologic deficits [20]. Gambian children homozygote for the TNF2 allele, a variant of the TNF-alpha gene promoter region, has a relative risk of 7 for death or severe neurological sequelae due to CM [21]. A subsequent study confirmed the association of the TNF-308 A allele of the TNF promoter with CM [22]. Release of TNF may be triggered by the local deposition of IgE containing immune complexes (IC) through cross-linking of CD23 on monocytes/macrophages in cerebral capillaries [23].

Increased levels of serum IC's have previously been associated with vasospasm in subarachnoid haemorrhages [24]. IC levels are increased in CM and this may be related to reduced clearance because of interleukin-4 mediated downregulation of Fc-receptors [25]. An erythrocyte complement receptor type 1 (E-CR1) promoter allele associated with higher E-CR1 expression and hence enhanced clearance of IC's conferred protection against CM [26].

An interesting recent study found evidence of cerebral arteriolar vasospasm in the murine* Plasmodium berghei* ANKA model of CM with retinal features of vascular collapse very similar to the angiographic findings in humans with CM. The researchers increased survival of mice with CM by use of nimodipine, a blocker of the vasoconstriction mediating L-type voltage-gated calcium channel, which is used to prevent and treat vasospasm associated with perivascular inflammation in subarachnoid haemorrhage [27]. Further support for a role of cerebral arteriolar vasospasm in CM is provided by the fact that in murine and human CM there is a low bioavailability of nitric oxide, which is an important vasodilator [28].

Haemolysis in malaria also leads to consumption of nitric oxide which is bound to free haemoglobin and haemolysis releases erythrocyte arginase, an enzyme that metabolizes arginine. This reduces the amount of arginine available for production of NO [29]. Investigation of the association of the single nucleotide polymorphism in the neuronal nitric oxide synthase (nNOS) gene with cerebral malaria revealed that the polymorphism associated with low basal transcriptional activity (-84 G- -→A and 276C- -→T) was associated with an increased risk of cerebral malaria [30]. The single nucleotide polymorphism -1173C-→T in the nitric oxide synthase 2 promoter was associated with increased fasting urine and plasma nitric oxide metabolite concentrations in Tanzanian children and was associated with protection against cerebral malaria [31]. In the* P. berghei* ANKA murine model NO supplementation in the form of a NO donor prevented vasoconstriction and improved blood flow in pial vessels [32]. Nitric oxide production is partially downregulated by IL-12 and polymorphisms in the gene encoding the IL-12 p40 subunit associated with increased IL-12 levels led in homozygous form to decreased production of nitric oxide and increased mortality in Tanzanian children with CM but not with severe anaemia in Kenyan children [33]. High levels of erythropoietin, which has been shown to upregulate endothelial nitric oxide synthase, were associated with protection against neurological sequelae in African children with CM [34].

### 3.3. Neurocysticercosis

Infection of the central nervous system with the larval stage of* Taenia solium* (neurocysticercosis) is the most common helminthic infection of the CNS and a cause of stroke in areas where cysticercosis is endemic. Over 50% of patients with subarachnoid involvement have hereby features of vasculitis. Antiparasitic treatment has been associated with enhanced subarachnoid inflammation and delayed cerebral infarcts. The focal cerebral lesions found in the absence of changes on conventional angiograms and with normal transcranial Doppler ultrasound point to vasospasm in their aetiology [35].

## 4. Brain Injury

Evidence of traumatic cerebral vasospasm (TCV) has been detected in military personnel with blast injuries to the head. Patients with TCV were also likely to have an intracranial bleed, traumatic aneurysm, or significant cerebral lobar injury. As indicator of cerebral vasospasm they had elevated cerebral blood flow velocities for approximately 14 days in one study and TCV was associated with increased mortality [36].

A link between inflammation and TCV provided a study investigating risk factors for cerebral vasospasm in 46 adults and children with TCV: fever on admission was an independently strongly predictive risk factor for the development of TCV (OR 22.2 95% CI 1.9 to 256.8). Importantly patients with hypothermia on admission did not develop TCV [37]. Underlying mechanisms were explored in the newborn and juvenile pig model of brain injury which showed that cerebrospinal fluid concentration of ET-1 was increased and NMDA receptor mediated vasodilation decreased and more so in the immature brain [38]. In humans investigators observed that the loss of nitric oxide bioavailability and decreased sensitivity to NO after traumatic brain injury were found to be contributory to impaired cerebral vascular autoregulation.

## 5. Subarachnoid Haemorrhage

Since the demonstration of arterial narrowing in the syndrome of cerebral vasospasm of subarachnoid haemorrhage (SAH) in 1951 and the further emphasis in 1978 by Weir et al., it has been proven that SAH gives rise to arterial narrowing and in turn ischaemia, causing infarction and poor outcome. Most research into delayed deterioration after SAH has been conducted in concordance with this axiom, with the goal of interrupting this perceived chain of events [39].

Autopsy studies in nonoperated SAH patients reported that 80% of fatal cases showed widely scattered triangular, round, or laminar ischemic lesions in the cortex. An ongoing clinical multicenter study reported that, in 18 post-SAH patients, the occurrence of spreading depolarization with cortical spreading ischaemia was associated with delayed infarcts in brain computed tomography and magnetic resonance imaging. These findings indicate a new SAH-related mechanism of acute brain injury involving the release of oxyhaemoglobin and ET-1, and NO deprivation, all of which are found in SAH and can cause cortical spreading depression and ischaemia in experimental models [40]. Endothelium-dependent dilation hereby seems to be reduced, partly because of reduced expression of guanylate cyclase [41]. Experimentally, neurogenic and endothelium-dependent vasodilatation have been shown to be altered by the presence of haemoglobin (Hb) in the bath [42]. A more complete review of this mechanism can be found elsewhere [43]. Cerebral vasomotor disturbances have been demonstrated in humans after SAH in measurements of cerebral blood flow by 133-xenon tomography. One of the most important functional consequences of SAH is the relative loss of cerebrovascular dilatory capacity after acetazolamide injection [44]. The sources of NO in the cerebral arteries [45] are such that it has been supposed that Hb scavenging of NO could induce vasospasm (VS). The prolonged presence of a perivascular clot seems necessary to maintain VS, since clot removal up to 3 days after haemorrhage reduces VS, but after 5 days, it has little influence [46].

Prostaglandin E2 (PGE2) is the eicosanoid molecule present in the highest concentration in the CSF after SAH. The concentration increases by more than 25 times soon after the haemorrhage and remains high for several days [47]. This rise probably explains the fever usually found in SAH. The production of PGE2 is induced by cyclooxygenase 2 induction by inflammatory mediators including TNF in platelets and microvessel endothelial cells [48, 49]. There is evidence of early increased TNF-concentration in the CSF of patients with SAH [47, 50]. PGE2 has been shown to induce contraction in large cerebral arteries [51].

## 6. The Role of Inflammatory Mediators in Generation of Vasospasm

*The Interaction of Inflammatory Mediators with Cerebral Vasculature.* To enable an understanding of the pathomechanisms involved in inflammation related cerebral vasospasm and resulting brain ischaemia it is important to analyse the interaction of inflammatory mediators with each other and the cerebral vasculature. A mediator, which was found to be of key importance in reduction of the risk of cerebral vasospasm in bacterial meningitis, cerebral malaria, brain injury, and subarachnoid haemorrhage, is nitric oxide (NO).

NO can be inactivated by reaction with the superoxide anion resulting in the formation of peroxynitrite. Inactivation of NO by superoxide anion may contribute to impaired NO-mediated dilatation of cerebral blood vessels under conditions in which reactive oxygen species are produced like brain injury and meningitis.

Production of superoxide anions has also been reported to occur in brain in response to seizures, fluid-percussion injury, perivascular blood, and ischaemia with reperfusion. Endothelial NO synthase is downregulated by TNF and NO by a negative-feedback mechanism [52, 53]. In addition to inflammatory mediators sensory fibres originating in the trigeminal ganglion modulate constrictor responses in the cerebral microcirculation in conditions like meningitis [7].

The most important factor leading to vasoconstriction in cerebral blood vessels is ET-1, a 21 amino acid peptide with three isopeptides produced by separate genes. Only ET-1 is produced in cerebral endothelium and mediates cerebral vasoconstriction via endothelin-A (ETA) receptors. ETA is localized in vascular smooth muscle cells, and stimulation leads to increase in intracellular calcium concentrations leading to vasoconstriction. ET-1 expression is enhanced by transforming growth factor-beta, haemoglobin, interleukin-1, and TNF and can be inhibited by NO, nitric oxide donor drugs, and dilator prostanoids via an increase in cellular cGMP and natriuretic peptides via an increase in cAMP levels. NO can produce relaxation of some blood vessels from some species via activation of potassium channels [7].

Cerebral blood vessels can produce 20-hydroxyeicosatetraenoic acid from arachidonic acid via a P-450 enzyme. This substance is a potent vasoconstrictor, which may produce this effect by inhibition of activity of potassium channels [7].

A further potential mechanism involved in cerebral vasospasm in cerebral inflammation is inhibition of potassium channels. Nicorandil, which produces relaxation of cerebral vessels by activation of potassium channels, partially reverses vasospasm in an experimental model of subarachnoid haemorrhage.

The effectiveness of other synthetic activators of potassium channels like aprikalim and cromakalim and CGRP indicate that activators of potassium channels in vascular muscle may help in cerebral vasospasm in cerebral inflammation [7].

The etiological role of specific cytokines in vasospasm in bacterial meningitis in vivo was highlighted by a study which documented a significant increase in IL-1-beta and IL-6 concentrations in patients with elevated cerebrovascular blood flow indicative of vasospasm in humans [14, 54]. A similar effect on vasospasm of IL-1 and IL-6 was suggested by a study of cerebral blood flow in subarachnoid haemorrhage where increased cerebral blood flow velocities were associated with significantly increased CSF concentrations of IL-1, IL-6, and TNF. High concentrations of these immunomediators in cerebrospinal fluid (CSF) may exert vasoactive effects; for example, IL-1 (MW 17 000 Da) or IL-6 (MW 26 000 Da) could easily get access to the walls of contiguous basal arteries from their adventitial side as even larger molecules (e.g., horseradish peroxidase, MW 40 000 Da) pass from the cisterna magna through the vessel wall to the basal membrane within minutes. This is possible because, by contrast with other arteries, the surface of the major cerebral arteries is not confined by collagen or fibroblasts but is in direct contact with the CSF.

**Figure 1:**
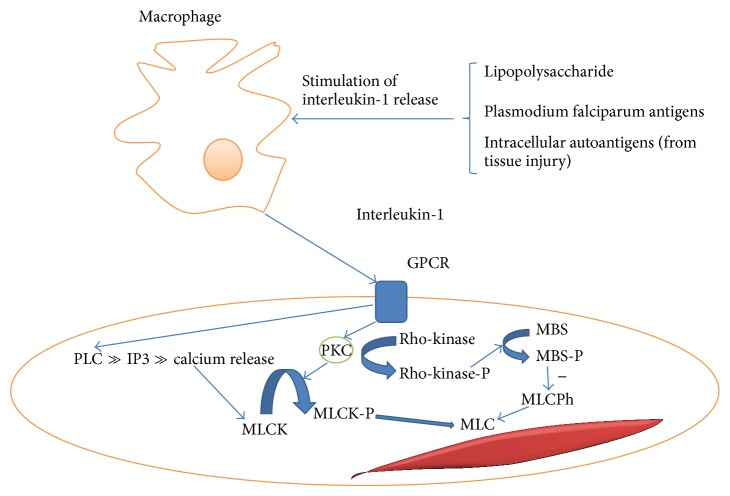
Mechanism for induction of cerebral vasospasm by interleukin-1 (GPCR = G-protein coupled receptor, PKC = protein kinase C, MLCK = myosin light chain kinase, MLC = myosin light chain, MBS = myosin-binding subunit of myosin light chain phosphatase, MLCPh = myosin light chain phosphatase, -P indicates phosphorylated state, PLC = phospholipase C, and IP3 = phosphatidylinositol trisphosphate).

An investigation in patients with SAH showed that levels of IL-1-beta but not TNF-alpha were increased and correlated with the later development of vasospasm. IL-1 acts through G-protein coupled receptors in three ways which cause contraction of vascular smooth muscle (see Figure 1).

1. Activation of phospholipase C generated phosphatidylinositol trisphosphate causes calcium release which leads to myosin light chain kinase activation leading to myosin light chain activation and calcium dependent vasospasm. Phosphorylation of myosin light chain (MLC) by MLC kinase is one of the most important steps for vascular smooth muscle contraction.
2. Activation of protein kinase C (PKC) through phospholipase C generated diacylglycerol (from phosphatidylinositol (4,5) bisphosphate) can act through phosphorylation and hence activation of the myosin light chain kinase. Prolonged contraction in cerebral vasospasm for up to two weeks can be mediated by this mechanism.
3. PKC activation regulates MLC phosphorylation through activation of rho-kinase and the myosin-binding subunit (MBS) of MLC phosphatase (MLCPh). Rho-kinase hereby phosphorylates MBS, which results in the inhibition of MLCPh [55]. The reduced MLCPh activity leads to increased phosphorylation and hence contractility of the MLC resulting in calcium independent vasospasm. Investigations in a porcine model revealed that hydroxyfasudil, a specific rho-kinase inhibitor, exerted an inhibitory effect on vasospasm both in vitro and in vivo. Western blot analysis showed that, during serotonin-induced contractions, the extent of phosphorylation of the MBS was significantly greater in the spastic than in the control segment. There was a highly significant correlation between the extent of MBS phosphorylation and contractions [55].

Another key mechanism whereby IL-1 mediates vasospasm may be through platelet derived growth factor. The receptors for this growth factor are also known to have tyrosine kinase activity and inhibition of tyrosine kinase is known to reduce vasospasm in a swine model [56, 57]. This action influences vascular reactivity to different agonists and might be crucial for the regulation of cerebral artery tone during vasospasm [58].

Calcium independent vasospasm may also be inducible by haemoglobin released in trauma or subarachnoid haemorrhage.

Haemoglobin might cause contraction of major cerebral arteries by scavenging nitric oxide, reducing neuronal nitric oxide synthase (NOS) in the adventitia of the cerebral arteries, and evoking dysfunction of endothelial NO synthase. A decrease in nitric oxide availability might produce prolonged pathological contraction, regardless of the concentration of intracellular calcium. Much of the work investigating the genetic predisposition to cerebral vasospasm focused on the endothelial isoform of nitric oxide synthase. Hemoglobin also stimulates the production of endothelins and the imbalance of nitric oxide and endothelin could be an important cause of pathological arterial contraction. In gene transfer experiments eNOS overexpression in animal and human intracranial arteries is vasoprotective after aneurysmal SAH [59]. Several investigations confirmed the association of the T-786C genotype of eNOS with vasospasm. For a summary of key mediators in cerebral vasospasm, their function, and disease studied see Table 1.

## 7. Therapeutic Approaches to Prevention and Correction of Vasospasm Associated with Cerebral Inflammation Tested in Clinical Trials in Humans

The most extensive experience with treatment approaches to vasospasm in cerebral inflammation has been gathered in the context of management of SAH. Clinical trials in humans have been reported for endothelin-1 antagonists, calcium channel blockers, including the cisternal placement of controlled-release nicardipine, intravenous magnesium sulphate, oral statins, rho-kinase inhibitors, tissue plasminogen activator, tirilazad, erythropoietin, and methylprednisolone. The evidence from controlled trials has been summarized in Table 2.

### 7.1. Endothelin-1A Receptor Antagonists

The selective endothelin-1A receptor antagonist clazosentan was assessed in a randomized placebo controlled trial (CONSCIOUS-1) and led to a significant reduction of angiographic cerebral vasospasm in SAH but without reduction in the number of cerebral ischaemia events. Subsequent phase II trials (CONSCIOUS-2 and CONSCIOUS-3) in SAH did not demonstrate any effect on morbidity or mortality. A meta-analysis of 4 RCTs with 2024 participants confirmed this finding but showed a reduction in delayed ischemic neurological deficit with the endothelin-1A receptor antagonist [67].

### 7.2. Calcium Channel Blockers

Calcium antagonists have the potential to prevent the occurrence of calcium dependent vasospasm and delayed ischemic neurological deficit by reducing calcium release (see Figure 1). They have been applied directly to the subarachnoid space [74]. Intraoperative implantation of nicardipine prolonged-release implants, so-called pellets into the basal cistern in close contact to the proximal cerebral vasculature, has been reported to reduce the occurrence of cerebral vasospasm and cerebral infarction and lead to an improvement in clinical outcome in SAH [75]. The magnesium ion is an antagonistic competitor for calcium binding sites, thus having the potential to reduce calcium dependent vasospasm, and has been used in the form of intravenous magnesium sulphate in a number of trials (see Table 2).

### 7.3. Tissue Plasminogen Activator

A recent meta-analysis of five randomized controlled trials (465 patients) of tissue plasminogen activator (tPA) or urokinase applied intrathecally reduced development of poor outcome and delayed cerebral ischaemia and vasospasm [70]. The mechanism proposed for this effect is probably not related to thrombolysis but to a direct effect of tPA on vascular tone. It is clear that the effect of tPA is endothelial cell dependent and, more specifically, NO synthase dependent. tPA seems to bind to a receptor present in endothelial cells that mediates the stimulation of NO synthase activity and thus inhibits the vasoconstrictive effect of phenylephrine [76].

### 7.4. Statins

Statins can reduce vasospasm by upregulating endothelial NO synthase expression. A threefold increase in endothelial NO synthase mRNA, protein, and enzymatic activity has been demonstrated following statin treatment, resulting in an increase in cerebral blood flow. Statin treatment has attenuated cerebral vasospasm and prevented delayed ischemic deficits in a murine SAH model [77]. A previous systematic review of 4 studies with a total of 190 patients showed no statistically significant effect on vasospasm on transcranial Doppler studies, delayed cerebral ischaemia, or mortality [78], but a more recent review demonstrated a reduction in mortality and delayed ischemic neurological deficits [66].

### 7.5. Rho-Kinase Inhibitor

The specific rho-kinase inhibitor fasudil hydrochloride was in a recent systematic review of 8 studies (843 patients) shown to reduce the occurrence of cerebral vasospasm and cerebral infarction significantly and led to an improvement in clinical outcome [71]. The mechanism is apart from a contribution to inhibition of calcium independent vasospasm (see above) probably also an increase of endothelial nitric oxide synthase (eNOS) expression by stabilizing eNOS mRNA, which can contribute to an increase of NO level to enhance vasodilatation.

### 7.6. Other Treatments

Steroid treatment can inhibit cytokine expression and has thus the potential to reduce cytokine mediated calcium dependent and independent vasospasm. In tuberculous meningitis adjuvant treatment with corticosteroids has been indirectly associated with features of reduced vasculopathy, which may include vasospasm.

A large randomised controlled trial showed improved mortality in tuberculous meningitis with adjuvant corticosteroids but did not show a reduction in severe disability; however, an observational MRI study did suggest a reduction in strokes in patients treated with corticosteroids [79, 80]. An open-label study of aspirin in the prevention of stroke and mortality in tuberculous meningitis found a nonsignificant reduction in stroke and a significant reduction in mortality with a possible synergism with corticosteroids [81]. Mechanisms responsible for a potential effect may be a reduction of the vasoconstricting effect of PGE2. The 21-aminosteroid tirilazad is a lipid peroxidation inhibitor which inhibits free radical-induced lipid peroxidation (LP) by a combination of lipid peroxide scavenging and a membrane-stabilizing action that limits the propagation of LP reactions between a lipid peroxide and an adjacent polyunsaturated fatty acid. There has been no consistent evidence of benefit in animal or human studies. Erythropoietin may act through increase in expression of endothelial nitric oxide synthase, thus increasing NO bioavailability.

## 8. Conclusions and Perspectives for Future Developments

There is evidence for common pathways leading to vasospasm in all forms of cerebral inflammation involving interleukin-1, tumor necrosis factor, endothelin-1, and nitric oxide. Treatments addressing the pathophysiology of vasospasm in one form of cerebral inflammation may therefore apply to all others. Treatment of cerebral vasospasm has been mainly investigated in aneurysmal SAH. Agents for which there is likely to be clinical benefit are calcium antagonists, statins, and intrathecal plasminogen activators (see Table 2). The fact that there is calcium dependent and independent vasospasm in inflammation (see Figure 1) shows that a combination of calcium antagonists with agents interfering with calcium independent vasospasm including agents improving the bioavailability of nitric oxide is the way forward. Future trials need to investigate their effects in patients with all forms of cerebral inflammation leading to neurological deficits attributable to vasospasm. In vivo correlates for reduction of vasospasm in the peripheral circulation could be explored as a surrogate for reduction of vasospasm in the cerebral (micro) vasculature.

Strategies to improve microvascular recruitment, which have been explored in models of septicaemia, could potentially be applied to cerebral vasospasm. Distributive shock, which occurs during sepsis and septic shock, is associated with an abnormal distribution of microvascular blood flow. Vasospasm of part of the arterioles supplying the capillary is a mechanism involved in this maldistribution. The clinical introduction of new microcirculatory imaging techniques, such as orthogonal polarization spectral and side-stream dark-field imaging (OPS/SDF), has allowed direct observation of the microcirculation at the bedside. Images of the sublingual microcirculation during septic shock and resuscitation have revealed that the distributive defect of blood flow occurs at the capillary level [82].

In rat models of cecal ligation and puncture, investigators have used intravital video microscopy to demonstrate that sepsis is characterized by decreased microcirculatory flow velocity, an abundance of stopped-flow microvessels, increased heterogeneity of microcirculatory flow, and low density of perfused capillaries. As these microcirculatory flow alterations can occur in the absence of global hemodynamic derangements (e.g., absence of arterial hypotension), microcirculatory dysfunction largely reflects intrinsic events occurring in the microvessels like localized vasospasm [83]. Application of microcirculatory recruitment maneuver procedures has been shown to be effective in promoting microcirculatory blood flow in clinical studies using OPS/SDF imaging [84–87]. Fluid resuscitation in combination with the nitric oxide donor nitroglycerine was shown to recruit disturbed microcirculation following pressure guided resuscitation in septic shock patients.

It is important to note that application of colloids in patients with SAH was associated with worse outcomes compared to matched controls in a subgroup analysis of the CONSCIOUS-1 prospective randomized trial of clazosentan for the prevention of angiographic vasospasm [88]. Future research needs to investigate whether colloids can bind nitric oxide and make this vasodilator less available by binding it.

Regarding future therapeutic interventions increasing nitric oxide bioavailability erythropoietin and L-arginine, which increased bioavailability of nitric oxide in patients with malaria [89], need to be explored as adjunctive treatment in children with cerebral malaria in randomised controlled trials. In vitro the availability of L-arginine does not appear to be a rate limiting step for the activity of the NO synthase because its half-saturating concentration is less than 3% of the plasma and endothelial concentration of this substrate.

The nitric oxide donor intravenous sodium nitrite has been shown to prevent and reverse cerebrovascular spasm in primates and has been demonstrated to be safe for administration in humans [90].

Support of pump function by dobutamine therapy has been shown to improve microcirculatory flow independent of improvement of global hemodynamic parameters [91]. Fluid application, nitric oxide donors, and dobutamine therapy need to be explored in isolation but also as combination of interventions to improve cerebral perfusion in cerebral inflammation associated with vasospasm.

A potential role of nonsteroidal anti-inflammatory drugs was revealed by landmark experiments on dog basilar arteries. Arachidonic acid induced sustained contractions and these responses were markedly inhibited (95%) by meclofenamate and similarly by indomethacin but aspirin had no such effect [92]. In the rabbit basilar artery model of cerebral vasospasm local intracranial delivery of ibuprofen accomplished using controlled-release polymers prevented vasospasm when administered within 6 hours but not at either 12 or 24 hours [93]. Delivery of ibuprofen via controlled-release polymers into the subarachnoid space of monkeys with subarachnoid haemorrhage prevented angiographic vasospasm after SAH as evident from patency of the middle cerebral artery [94].

Piroxicam was more effective than the above-mentioned drugs in the SAH dog model after angiographic analysis and analysis of behavioural changes [95]. Early studies using prostacyclin found that isolated canine basilar arteries contraction induced by prostaglandin E2, hemoglobin, or serum was relaxed by prostacyclin but not indomethacin [96]. In isolated human pial arteries prostacyclin relaxed human pial arteries contracted by either PGF2 alpha, noradrenaline, serotonin, or haemorrhagic CSF [97]. In an animal study meloxicam reduced ultrastructural and morphometric vasospastic changes in rats with subarachnoid haemorrhage [98]. Investigation comparing rate of subsequent permanent disability from vasospasm and angiographic and delayed cerebral ischaemia in patients with SAH with and without aspirin intake found no association with poor outcome [99, 100].

There are new areas of pathophysiology research where the role of vasospasm needs to be explored thoroughly in order to find avenues for therapeutic interventions. Investigations could evaluate whether intraventricular haemorrhage occurring commonly in the neonatal period in preterm neonates is associated with vasospasm increasing morbidity. Case reports of intraventricular haemorrhage from AV malformations and aneurysms in adults demonstrated vasospasm [101, 102].

Future research needs to explore the usefulness of a number of agents, which have been demonstrated to reduce vasospasm in animal models including L-citrulline [103], janus kinase-2 [104], endothelin converting enzyme inhibitors [105], caspase inhibitor Z-VAD-FMK [106], anti-E selectin monoclonal antibodies [107], trehalose [108], and curcumin, which was demonstrated to protect against the development of cerebral vasospasm and secondary cerebral infarction after subarachnoid haemorrhage in mice by its broad anti-inflammatory activity including limitation of superoxide generation and influence on inducible nitric oxide synthase expression [109].

Aggressive maintenance of normothermia or mild hypothermia in patients with military blast injury resulted in a dramatic reduction in the incidence of posttraumatic vasospasm from 47.5% to 5%. Future studies need to confirm this finding and investigate whether hypothermia can reduce cerebral inflammation related ischemic damage to the brain in other conditions like bacterial meningitis or cerebral malaria [37].

## Figures and Tables

**Table 1:** Inflammatory mediators and their role in cerebral vasospasm.

Mediator	Function of mediator	Disease model where role has been established	References
(i) Nitric oxide	(i) Vasodilator (ii) Inhibitor of endothelin-1 expression	(i) Cerebral malaria (ii) Bacterial meningitis (iii) Subarachnoid haemorrhage	[1, 7, 16, 28, 32, 41, 45]

(i) Endothelin-1	(i) Vasoconstrictor	(i) Bacterial meningitis (ii) Subarachnoid haemorrhage	[7, 15]

(i) Interleukin-1 (ii) Interferon gamma (iii) Tumor necrosis factor	(i) Activators of protein kinase C (ii) Stimulators of release of endothelin-1	(i) Bacterial meningitis (ii) Cerebral malaria (iii) Subarachnoid haemorrhage	[7, 14, 56]

**Table 2:** Medication for treatment of cerebral vasospasm: the evidence from controlled trials in humans.

Intervention	Type of study	Pathology	Results: odds ratio (OR) or relative risk (RR) for occurrence in intervention group compared to group without intervention	Reference
Calcium channel blockers	Systematic review (SR) (six randomized controlled trials (RCTs))	Acute brain injury	Death: OR 0.91 [95% 0.70–1.17] Death and severe disability OR 0.85 [95% 0.68 to 1.07] In the subgroup of traumatic subarachnoid haemorrhage patients, pooled odds ratio was 0.59 [95% CI 0.37–0.94]; three RCTs reporting death and severe disability as an outcome in this subgroup: OR 0.67 [95% CI 0.46–0.98]	[60]

Calcium channel blockers	SR (16 RCTs)	Aneurysmal subarachnoid haemorrhage (aSAH)	Overall, calcium antagonists reduced the risk of poor outcome: the relative risk (RR) was 0.81 [95% CI 0.72 to 0.92]; the corresponding number of patients needed to treat was 19 [95% CI 1 to 51]; for oral nimodipine alone the RR was 0.67 [95% CI 0.55 to 0.81]; for other calcium antagonists or intravenous administration of nimodipine the results were not statistically significant; calcium antagonists reduced the occurrence of secondary ischaemia and showed a favourable trend for case fatality; for magnesium, in addition to standard treatment with nimodipine, the RR was 0.75 [95% CI 0.57 to 1.00] for a poor outcome and 0.66 [95% CI 0.45 to 0.96] for clinical signs of secondary ischaemia	[61]

Nicardipine	SR (five controlled trials)	aSAH	Risk of poor outcome (death, vegetative state, or dependency) OR: 0.58 (95% CI 0.37–0.9) Mortality odds ratio: 0.45 [95% CI 0.15 to 1.29]	[62]

Magnesium sulfate	Prospective randomised study	Eclampsia	Significant reduction of pulsatility index (*P* = 0.002) and mean flow velocity in middle cerebral artery (*P* = 0.02)	[63]

Prophylactic magnesium sulfate	SR of ten randomized, parallel group controlled trials	aSAH	Glasgow outcome scale and modified Rankin scale RR: 0.93 (95% CI 0.82 to 1.06), mortality: 0.95 (95% CI 0.76–1.17), delayed cerebral ischaemia RR: 0.54 (95% CI 0.38 to 0.75), delayed ischemic neurological deficit (DIND): RR of 0.93 [95% CI 0.62–1.39], transcranial Doppler vasospasm: RR: 0.72 [95% CI 0.51–1.03]	[64]

Induced hypermagnesaemia	RCT	aSAH	Vasospasm on digital subtraction angiography OR: 0.51, [95% CI, 0.26 to 1.02], neurological recovery OR for worse outcome: 0.71 [95% CI 0.39 to 1.32]	[65]

Statins	SR (six RCTs)	aSAH	Incidence of vasospasm RR: 0.80, [95% CI: 0.54–1.17], poor neurological outcome: RR: 0.94 [95% CI, 0.77 to 1.16] Mortality: RR: 0.30 [95% CI, 0.14–0.64] DIND: RR: 0.58 [95% CI: 0.37–0.92]	[66]

Endothelin receptor antagonists	SR (four RCTs)	aSAH	Incidence of DIND: relative risk: 0.8 [95% CI 0.67–0.95], angiographic vasospasm RR 0.62, [95% CI 0.52 to 0.72], unfavourable outcome: RR: 0.87, [95% CI 0.74–1.02], mortality RR 1.05 [95% CI 0.77 to 1.45]	[67]

Endothelin-1A receptor antagonist Clazosentan	SR (four RCTs)	aSAH	Relative risk for incidence of DINDs: 0.76 (95% CI 0.62–0.92), delayed cerebral infarction: 0.79 [95% CI 0.63–1.00], Glasgow outcome scale extended RR: 1.12 [95% CI 0.96–1.30], mortality RR: 1.02 [95% CI 0.70–1.49]	[68]

Intravenous methylprednisolone	RCT	aSAH	Symptomatic vasospasm 26.5% in intervention group compared with 26% in placebo group, functional outcome scale reduced in the methylprednisolone group with risk difference 19.3% [95% CI 0.5–37.9%]; outcome poor in 15% of patients in the methylprednisolone group versus 34% in the placebo group	[69]

Tissue plasminogen activator	SR (five RCTs)	aSAH	Overall, use of intrathecal thrombolytics was associated with significant reductions in the development of poor outcomes (OR 0.52, 0.34–0.78, *P* < 0.01), DINDs (OR 0.54, 0.34–0.87, *P* = 0.01), and angiographic vasospasm (OR 0.32, 0.15–0.70, *P* < 0.01)	[70]

Rho-kinase inhibitor	SR (8 controlled trials)	aSAH	Absence of symptomatic vasospasm, occurrence of low density areas associated with vasospasm on CT, and occurrence of adverse events were similar between the two groups; the clinical outcomes were more favorable in the fasudil group than in the nimodipine group (*P* = 0.04)	[71]

Tirilazad	SR (five RCTs)	aSAH	There was no significant difference between the two groups at the end of follow-up for the primary outcome, death: OR: 0.89, [95% CI 0.74 to 1.06], or in poor outcome (death, vegetative state, or severe disability) OR 1.04 [95% CI 0.90 to 1.21]; fewer patients developed delayed cerebral ischaemia in the tirilazad group than in the control group OR 0.80 [95% CI 0.69 to 0.93]; subgroup analyses did not demonstrate any significant difference in effects of tirilazad on clinical outcomes	[72]

Erythropoietin	RCT	aSAH	No differences were demonstrated in the incidence of vasospasm and adverse events; patients receiving EPO had a decreased incidence of severe vasospasm from 27.5 to 7.5% (*P* = 0.037), reduced DIDs with new cerebral infarcts from 40.0 to 7.5% (*P* = 0.001), a shortened duration of impaired autoregulation (ipsilateral side, *P* < 0.001), and more favorable outcome at discharge (favorable Glasgow outcome scale score, *P* = 0.039); among the 71 survivors, the EPO group had fewer deficits measured with National Institutes of Health Stroke Scale (median score 2 versus 6, *P* = 0.008)	[73]
